# A new species of the genus *Euxiphocerus* (Diptera, Dolichopodidae) from Korea with checklist and key to species of the genus

**DOI:** 10.3897/BDJ.12.e124067

**Published:** 2024-07-05

**Authors:** Young-Kun Kim, Sang Jae Suh, Dongmin Kim

**Affiliations:** 1 School of Applied Biosciences, Kyungpook National University, Daegu, Republic of Korea School of Applied Biosciences, Kyungpook National University Daegu Republic of Korea; 2 Department of Plant Medicine, Kyungpook National University, Daegu, Republic of Korea Department of Plant Medicine, Kyungpook National University Daegu Republic of Korea; 3 Institute of Plant Medicine, Kyungpook National University, Daegu, Republic of Korea Institute of Plant Medicine, Kyungpook National University Daegu Republic of Korea; 4 Department of Applied Biology, Kyungpook National University, Daegu, Republic of Korea Department of Applied Biology, Kyungpook National University Daegu Republic of Korea

**Keywords:** *Euxiphoceruslignicola* sp. nov., Systenini, Medeterinae, Palearctic region

## Abstract

**Background:**

*Euxiphocerus* Parent, 1935 is a small genus consisting of three species only from the Afrotropical region.

**New information:**

During a survey of the long-legged flies from Korea, the authors discovered a new species, *Euxiphoceruslignicola* **sp. nov.** Detailed morphological characters and photographs of the new species, as well as a checklist and key to species of this genus, are provided herein. The two species described from Oriental China, *Systenusjinxiuensis* Lin & Yang, 2022 and *S.sinensis* Yang & Gaimari, 2004, are transferred to *Euxiphocerus* Parent, 1935 (**comb. nov.**) by similarity in external morphological characters.

## Introduction

To date, the genus *Euxiphocerus* Parent, 1935, has consisted of three species only from the Afrotropical region (Grichanov 2009). This genus was erected by Parent (1935) with the type species *E.wulfi* Parent, 1935, and it was previously placed within the subfamily Rhaphiinae. Later, Ulrich (1981) included this genus in the subfamily Systeninae, which was designated by Robinson (1970) with the monotypic genus *Systenus* Loew, 1857. Subsequently, Bickel (1986) combined Systeninae with Medeterinae but did not mention the position of the genus *Euxiphocerus*. Following this, Grichanov (2009) proposed the tribe Systenini as a new status within the subfamily Medeterinae, placing the genus *Euxiphocerus* under this tribe along with the genus *Systenus*.

**Figure 1.**
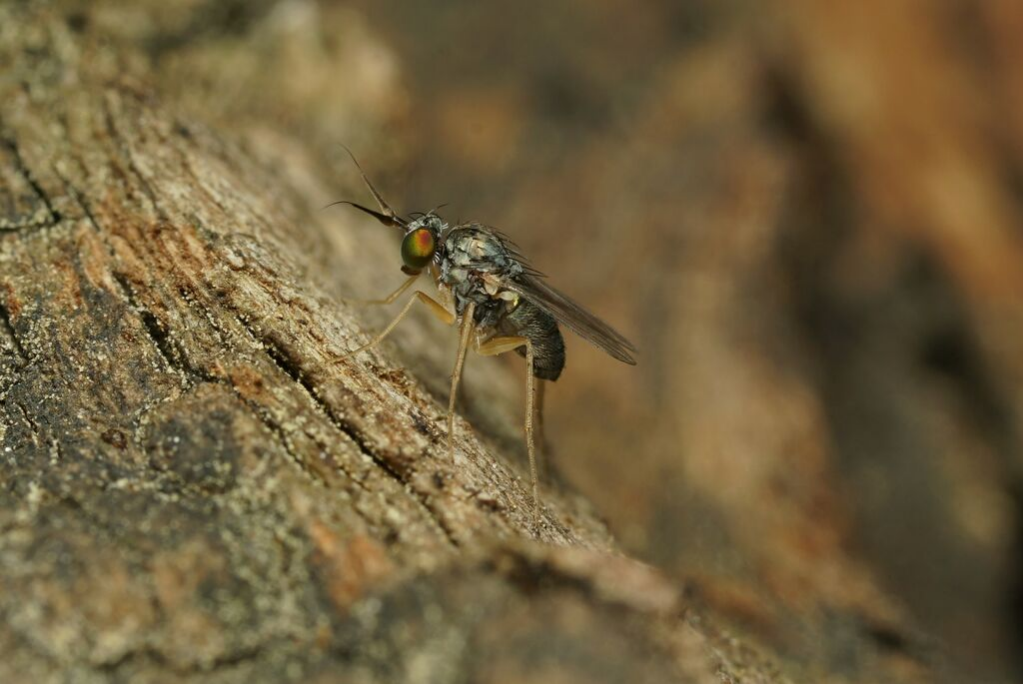
*Euxiphoceruslignicola* sp. nov. male on tree trunk.

**Figure 2.**
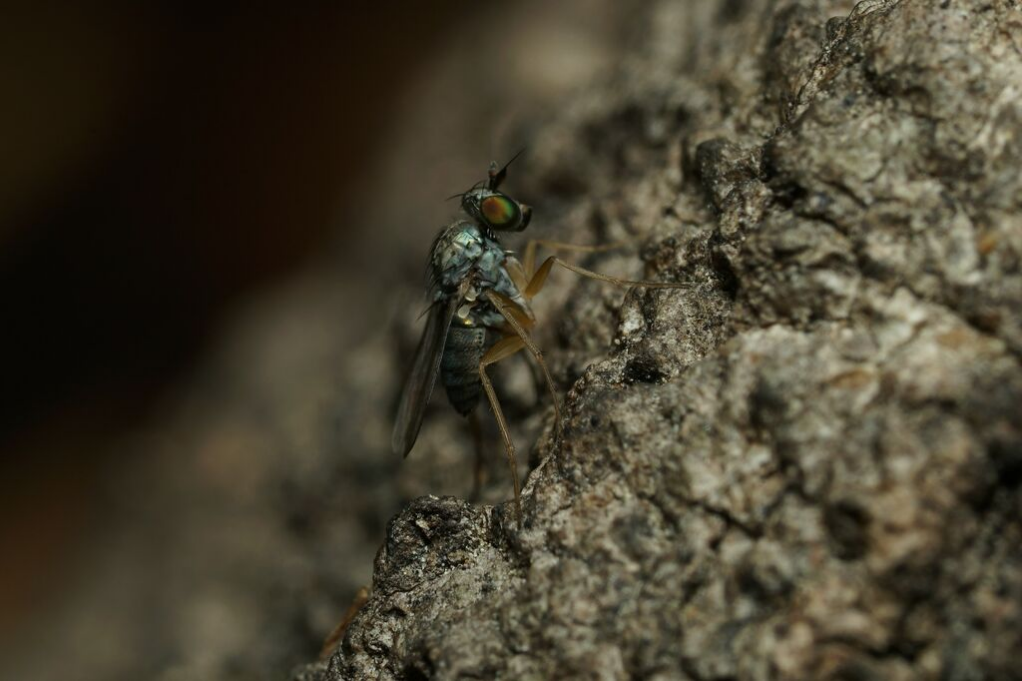
*Euxiphoceruslignicola* sp. nov. female on tree trunk.

During a survey of the long-legged flies from Korea, authors observed a new *Euxiphocerus* species on tree trunks and in tree holes (Figs 1, 2) So far, the ecology and biology of the species of this genus are unknown, but they are believed to be associated with tree trunks, similar to *Systenus* species.

In addition, two species described from Oriental China, *S.jinxiuensis* Lin & Yang, 2022 and *S.sinensis* Yang & Gaimari, 2004, are transferred to *Euxiphocerus* Parent, 1935 (comb. nov.), having similar morphological features: male elongated postpedicel (5–6 times longer than width), short male 7th abdominal segment, a pair of large and a pair of small epandrial lobes, and a deeply bifurcated surstylus in males.

## Materials and methods

External features, including male genitalia, were photographed using an Olympus SZX 16 stereomicroscope (Olympus, Tokyo, Japan), Olympus BX50 compound microscope, Olympus DP25 camera, and Michrome 16 CMOS camera (Tucsen, Fujian, China). For observation, the dissected male terminalia was heated in 10% potassium hydroxide (KOH) solution at about 80°C for 20–25 min. A series of images were montaged using Helicon Focus (HeliconSoft, Kharkiv, Ukraine). All images were further processed using Adobe Photoshop. All specimens examined in the present study were deposited in the collection of the Department of Plant Medicine at Kyungpook National University, Daegu, Korea. Morphological terms followed Cumming and Wood (2017). Abbreviations used in the manuscript are as follows: **AF** = Afrotropical region; **OR** = Oriental region; **PA** = Palearctic region.

## Taxon treatments

### Euxiphocerus

Parent, 1935

FBDF97B1-47AE-54C8-ABEF-92283D3F51FC

Euxiphocerus Parent, 1935: 122. Type species: *Euxiphoceruswulfi* Parent, 1935 (monotypy).

#### Diagnosis

Occiput is weakly convex backward. Compound eyes have tiny setulae between facets. Male Compound eyes are narrowly separated on face. Male face is narrow, narrowing downward. Vertex is not excavated. Upper occiput is distinctly concave. Vertical seta is nearly at level of oculus. Postocular setae are one-rowed and flattened. Male antennal pedicel is greatly reduced. Male postpedicel is 5–6 times longer than high at base. Arista-like stylus is apical. Mesonotum has flat mid-posterior slope. Posterior pair of acrostichal setae are distinctly larger than preceding pairs and offset laterally. 6 strong dorsocentral setae are decreasing in size anteriorly. Proepisternum is not haired, only with separate setae on mid-lower portion. Setulae on legs are uniformly short. Hind coxa has 1 outer seta at middle. Mid and hind femora do not have anterior preapical setae. R_4+5_ and M_1_ are subapically bowed. Distal sector of R_4+5_ and M_1_ have flexion. Anterior cubital cell is absent. Cua+CuP vein is weak. Male abdominal segment 6 is large triangular-shaped, with setae and setulae. Male 7th abdominal segment is short and has distinct tergite and sternite. Male genitalia are mostly exposed. Male hypopygium is sessile, with a pair of large and a pair of small epandrial lobes, with broad and deeply divided dorsal and ventral arms of surstylus. Apical subepandrial processes are absent and postgonite is indistinct. Male cercus usually thickened basally (based on Grichanov 2009).

### Euxiphocerus lignicola sp. nov.

E5531EB0-50B5-54F0-AFF5-0F4E8130E2F1

2A4FE108-7F14-4B79-9049-9DA5BC2A6E3D

#### Materials

- **Type status:** Holotype. **Occurrence:** sex: male; lifeStage: adult; occurrenceID: C1E90694-304B-5E3B-ACA1-E8B413107A10; **Location:** country: Korea; countryCode: Korea/KR; stateProvince: Daegu; county: Buk-gu; municipality: Sangyeok-dong; locality: KNU campus; verbatimLatitude: 35°53'24.7"N; verbatimLongitude: 128°36'29.9"E; **Event:** year: 2018; month: 8; day: 3
- **Type status:** Paratype. **Occurrence:** sex: male; lifeStage: adult; occurrenceID: ADE17B77-5947-5042-83A3-5E56ACC7B383; **Location:** country: Korea; countryCode: KR; stateProvince: Daegu; county: Buk-gu; municipality: Sangyeok-dong; locality: KNU campus; verbatimLatitude: 35°53'24.7"N; verbatimLongitude: 128°36'29.9"E; **Event:** year: 2016; month: 7; day: 22
- **Type status:** Paratype. **Occurrence:** sex: 2 males, female; lifeStage: adult; occurrenceID: AE02C3D5-7823-5B9C-B54D-F97080D615DD; **Location:** country: Korea; countryCode: KR; stateProvince: Daegu; county: Buk-gu; municipality: Sangyeok-dong; locality: KNU campus; verbatimLatitude: 35°53'24.7"N; verbatimLongitude: 128°36'29.9"E; **Event:** year: 2019; month: 5; day: 24
- **Type status:** Paratype. **Occurrence:** sex: female; lifeStage: adult; occurrenceID: 6D363C7F-B058-521B-807B-6FD581CE6475; **Location:** country: Korea; countryCode: KR; stateProvince: Daegu; county: Dalseo-gu; municipality: Dowon-dong; locality: Mt.Sampilbong; verbatimLatitude: 35°48'20.7"N; verbatimLongitude: 128°32'37.6"E; **Event:** year: 2019; month: 9; day: 18
- **Type status:** Paratype. **Occurrence:** sex: male, 2 females; lifeStage: adult; occurrenceID: 213E5E3A-ED87-5819-A732-E1324DD469A7; **Location:** country: Korea; countryCode: KR; stateProvince: Daegu; county: Buk-gu; municipality: Sangyeok-dong; locality: KNU campus; verbatimLatitude: 35°53'24.7"N; verbatimLongitude: 128°36'29.9"E; **Event:** year: 2021; month: 6; day: 2
- **Type status:** Paratype. **Occurrence:** sex: female; lifeStage: adult; occurrenceID: CB33FDD9-B73E-5117-AEDA-6AFA3DBB1AA1; **Location:** country: Korea; countryCode: KR; stateProvince: Daegu; county: Dalseo-gu; municipality: Dowon-dong; locality: Mt.Sampilbong; verbatimLatitude: 35°48'20.7"N; verbatimLongitude: 128°32'37.6"E; **Event:** year: 2021; month: 7; day: 18
- **Type status:** Paratype. **Occurrence:** sex: male, female; lifeStage: adult; occurrenceID: 0B7E59BB-F021-5B22-8A6D-9CC6F12C1F77; **Location:** country: Korea; countryCode: KR; stateProvince: Daegu; county: Dalseo-gu; municipality: Dowon-dong; locality: Mt.Sampilbong; verbatimLatitude: 35°48'20.7"N; verbatimLongitude: 128°32'37.6"E; **Event:** year: 2021; month: 7; day: 25
- **Type status:** Paratype. **Occurrence:** sex: 2 males, 4 females; lifeStage: adult; occurrenceID: EB7AACB3-AB3B-5619-B47E-45BDF2FB0C30; **Location:** country: Korea; countryCode: KR; stateProvince: Daegu; county: Dalseo-gu; municipality: Dowon-dong; locality: Mt.Sampilbong; verbatimLatitude: 35°48'20.7"N; verbatimLongitude: 128°32'37.6"E; **Event:** year: 2023; month: 6; day: 18

#### Description

**Figure 3.**
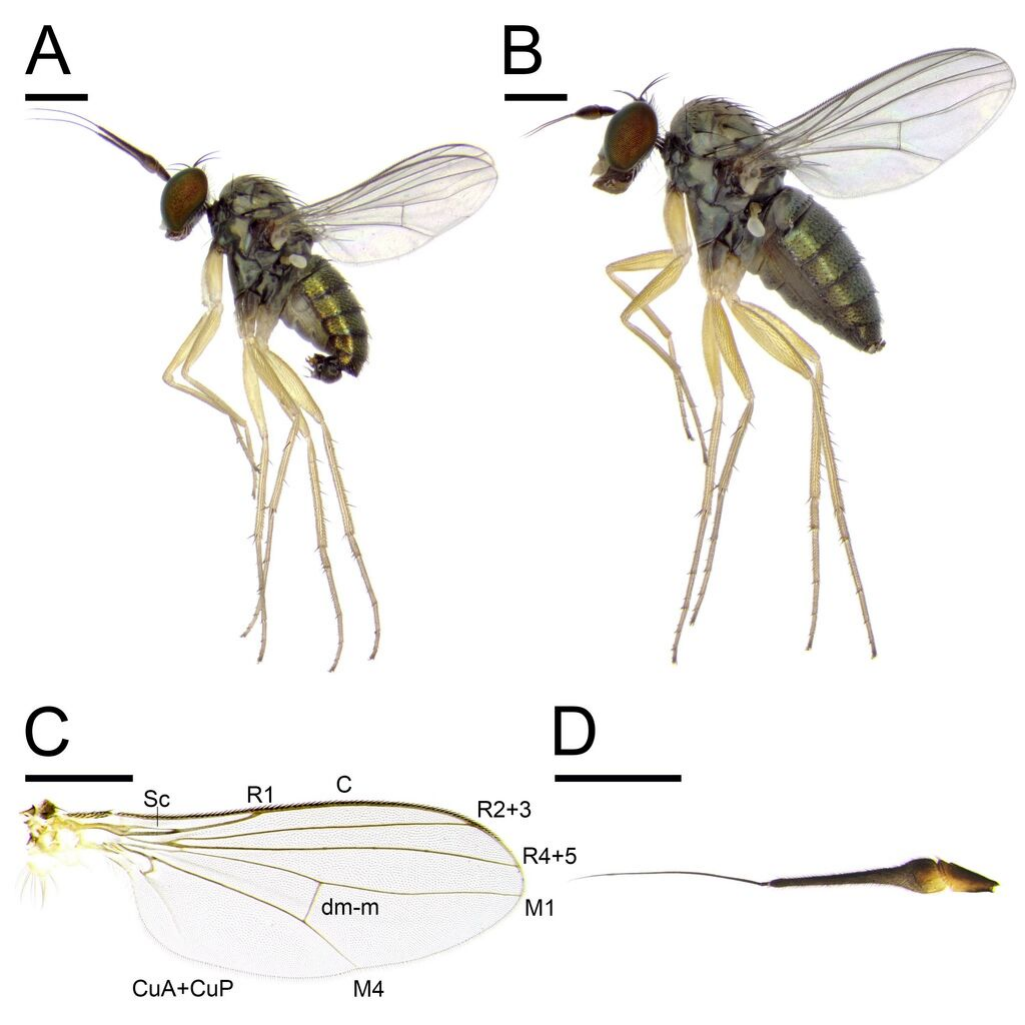
*Euxiphoceruslignicola* sp. nov.; **A** male, lateral view; **B** female, lateral view; **C** male wing; **D** male antenna, lateral view. Scale bars: 0.5 mm.

**Figure 4.**
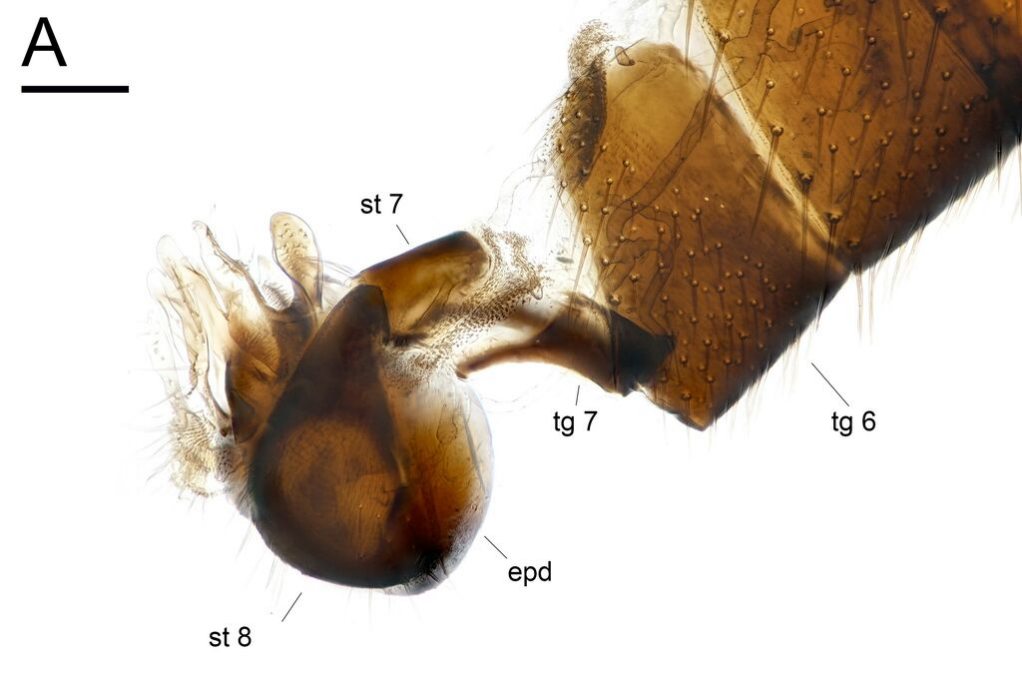
*Euxiphoceruslignicola* sp. nov.; **A** male abdomen, lateral view. Scale bar: 0.1 mm. Abbreviations: **epd** = epandrium; **st** = sternite; **tg** = tergite.

**Figure 5.**
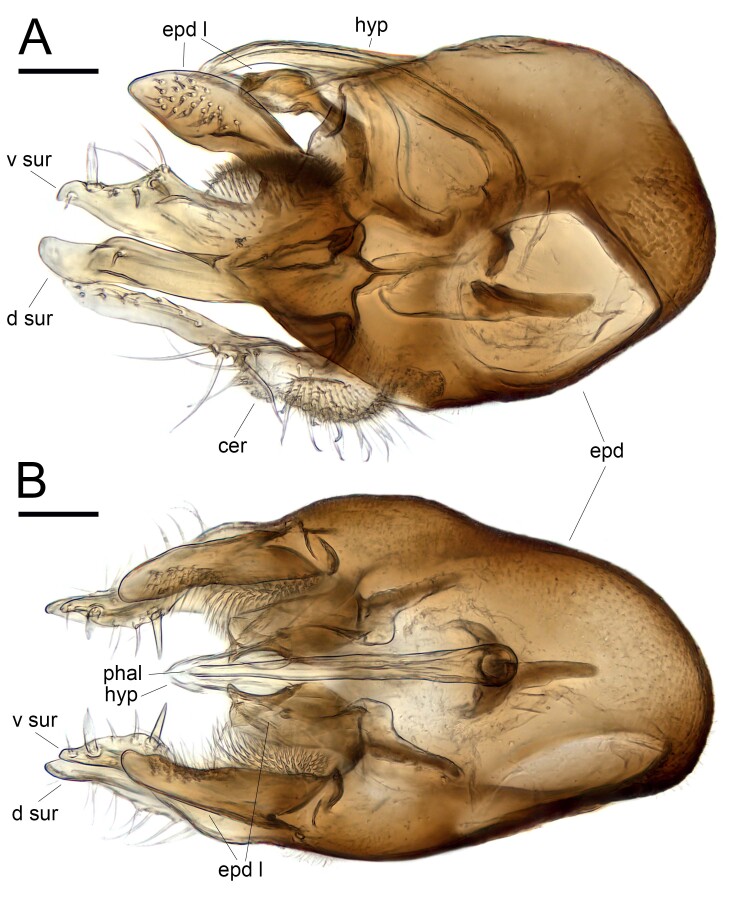
*Euxiphoceruslignicola* sp. nov.; **A** male genitalia, lateral view; **B** male genitalia, ventral view. Scale bars: 0.05 mm. Abbreviations: **d sur** = dorsal surstylus; **epd** = epandrium; **epd l** = epandrial lobe; **hyp** = hypandrium; **phal** = phallus; **v sur** = ventral surstylus.

Male (Figs 3, 4, 5). Body length: 2.6–2.8 mm. Wing length: 2.3–2.4 mm.

**Head**: metallic bluish green with mainly black setae and covered with white tomentum; one long ocellar seta reclinate and divergent; one short postocellar seta proclinate; one long vertical seta proclinate and convergent, and positioned slightly forward than ocellar seta line; one pale yellow postvertical seta convergent and proclinate; postocular setae pale yellow in a single row; face and clypeus covered with thick white tomentum; compound eye with tiny white setulae between facets; lower occiput with one long pale yellow seta and several pale yellow setulae; scape dark brown to pale brown without setae; pedicel pale brown with apical ring of setae; postpedicel mostly dark brown, pubescent and long, six times as long as basal width, and basal 1/5 swollen and ventrally pale orange; arista-like stylus brown, short pubescent, and slightly shorter than postpedicel; palpus pale yellow with pale yellow setulae; proboscis brown with pale setulae. **Thorax**: metallic bluish green with mainly dark brown setae and covered with white tomentum; 11–12 pale brown acrostichial setae biseriate; six dorsocentral setae; one long and two tiny postpronotal setae; one presutural and one postsutural intra-alar setae; one presutural and two postsutural supra-alar setae; two notopleural setae; one postalar seta; two marginal scutellar setae, dark brown median seta about twice as long as pale brown lateral seta; proepisternum with one long and 2–3 fine pale yellow setae. **Legs**: mainly pale yellow and covered with pale yellow setulae; fore coxa with white setae and setulae; fore femur without strong setae; fore tibia with one short brown posterodorsal seta; fore tarsus without strong setae; fore tarsomere 5 slightly brownish at apical half; relative ratio of fore femur, tibia and tarsomere 1–5: 8.8:9.0:3.3:1.3:1.0:0.8:1.0; mid coxa with brown basal half with white setae and setulae; mid femur without strong setae; mid tibia with one anterodorsal and two posterodorsal setae and apical ring of dark brown setae; mid tarsus without strong setae; mid tarsomere 5 slightly brownish at apical half; relative ratio of mid femur, tibia, tarsomere 1–5: 10.8:11.3:5.8:3.5:2.3:1.3:1.0; hind coxa with one white seta; hind femur without strong setae; hind tibia with one anterodorsal and five posterodorsal setae and apical ring of dark brown setae; hind tarsus without strong setae; hind tarsomere 5 slightly brownish at apical half; relative ratio of hind femur, tibia, tarsomere 1–5: 8.2:9.6:1.6:4.2:2.2:1.6:1.0. **Wings**: hyaline; veins mainly pale brown; C ended at M_1_; Sc fused to R_1_ at 1/3; R_1_ slightly curved to posterior; R_2+3_ almost straight; R_4+5_ slightly curved to posterior; M_1_ straight and slightly curved to anterior at 3/4; R_4+5_ and M_1_ slightly convergent at apex; M_4_ straight; crossvein dm-m straight; relative ratio of apical M_4_ to dm-m: 1.0:0.6; CuA+CuP fold-like and not reaching the wing margin; calypter pale yellow with pale yellow setulae on fringe; halter pale yellow. **Abdomen**: metallic bluish green with mainly pale yellow to pale brown setae and setulae and covered with white tomentum; posterior margin of tergites with relatively long setae; 7th tergite short, covered by 6th segment; epandrium dark brown, longer than wide with inner dense setulae between the surstylus and epandrial lobe; hypandrium fused to epandrium, simple; epandrial lobe broad, with dense setae at inner side; surstylus pale yellow and deeply bifurcated, dorsal lobe finger-like, ventral lobe almost same length as dorsal lobe with thick setae at inner side; cercus pale yellow and slender, basal 2/5 thick; phallus apically pointed.

**Figure 6.**
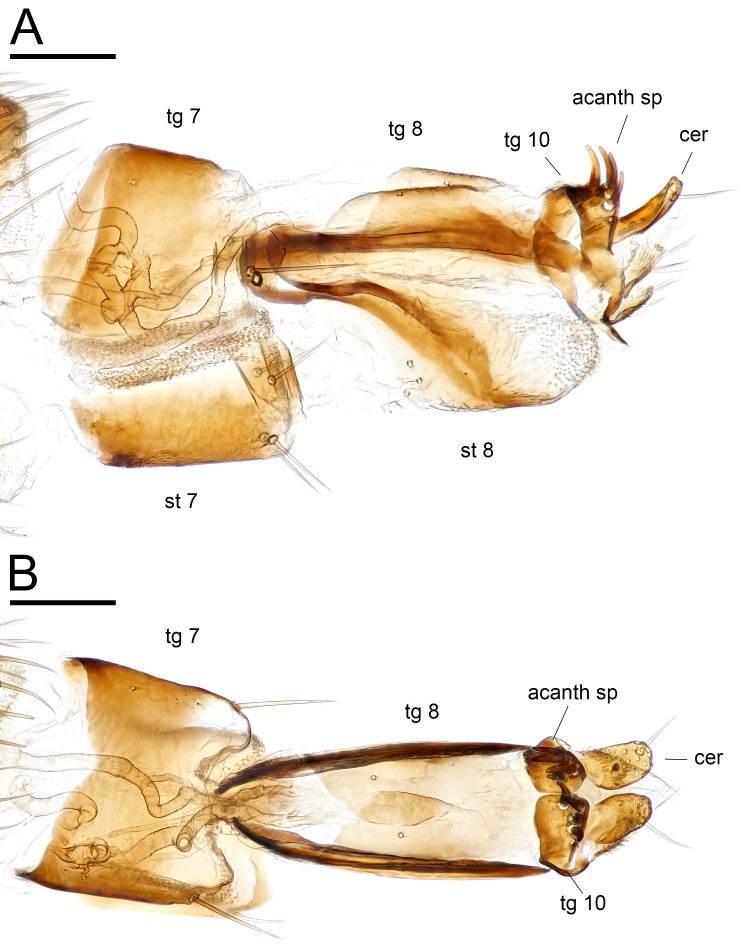
*Euxiphoceruslignicola* sp. nov.; **A** female genitalia, lateral view. **B** ditto, dorsal view. Scale bars: 0.1 mm. Abbreviations: **acanth sp** = acanthophorite spine; **cer** = cercus; **st** = sternite; **tg** = tergite.

**Female** (Figs 3, 6). Body length: 2.7–3.1 mm. Wing length: 2.4–2.7 mm. Almost the same as the male, except for some characteristics: postpedicel short, slightly longer than width; arista-like stylus about 2.5 times longer than postpedicel; palpus and proboscis larger than those in male; anterior half of tergite 8 dorsally spilited; tergite 10 with anterior one pair and a row of posterior 3–4 pairs of acanthophorite spines; cercus digitiform and arosed to upside with one ventral setula.

#### Diagnosis

Setae on head and thorax are mainly black and dark brown. Arista-like stylus without any ornament at apex. Fore and hind coxae are mainly yellow to pale yellow. All coxae have white setae. Setae on abdomen are mainly pale yellow to pale brown. Dorsal lobe of surstylus without ventral projection.

#### Etymology

The specific name is derived from the Latin words "*lignum*," meaning "wood" or "timber," and "*cola*" meaning "inhabitant" or "dweller."

#### Ecology

Species of the genus *Systenus* are known to be tree-trunk associated, and the larvae develop in tree-hole debris (Oboňa et al. 2012, Vaillant 1978). In this research, this new species was also observed on tree trunks and in tree holes inhabited by other *Systenus* spp. In this regard, *Euxiphocerus* and *Systenus* species are believed to have similar or the same ecologies.

#### Taxon discussion

This new species resembled *Euxiphocerussavannensiscapensis* Grichanov, 2009, but it can be differentiated by the following characteristics: coxae with white setae (*vs.* with black setae); abdomen with pale yellow setae (*vs.* with black setae); dorsal lobe of surstylus without ventral projection (*vs.* with ventral projection).

#### Notes

This is the first record of the genus *Euxiphocerus* from the PA.

### Euxiphocerus jinxiuensis

(Lin & Yang, 2022) comb. nov.

DB26E303-0AC5-5286-A34E-C9E4A67414C8

Systenus jinxiuensis Lin & Yang, 2022: 294 (Type-locality: China, Guangxi, Jinxiu).

#### Taxon discussion

This species has some similar morphological features of genus *Euxiphocerus*: elongated postpedicel (6 times longer than width), a pair of large and a pair of small epandrial lobes, and a deeply bifurcated surstylus in males.

### Euxiphocerus sinensis

(Yang & Gaimari, 2004) comb. nov.

CC0E48E2-FECB-5E5B-95C5-78B69AFF0C7C

Systenus sinensis Yang & Gaimari, 2004: 176 (Type-locality: China, Yunnan, Xishuangbanna).

#### Taxon discussion

This species has some similar morphological features of genus *Euxiphocerus*: elongated postpedicel (4.8 times longer than width), a pair of large and a pair of small epandrial lobes, and a deeply bifurcated surstylus in males.

## Checklists

### Checklist of the genus *Euxiphocerus* of the world

#### Euxiphocerus disjunctus

Grichanov, 2009

129967B4-FFB9-572A-B38D-46B6C97ECB36

Euxiphocerus disjunctus Grichanov, 2009: 129 (Type-locality: South Africa, E. Cape, Grahamstown).

##### Distribution

AF: Namibia, South Africa (Grichanov 2009).

#### Euxiphocerus lignicola

sp. nov.

DE485378-66F4-5E76-82C4-DABDD76B7C76

##### Distribution

PA: Korea.

#### Euxiphocerus savannensis capensis

Grichanov, 2009

36E25095-25EA-57E3-A8B6-FACB4C215110

Euxiphocerus savannensis capensis Grichanov, 2009: 131 (Type-locality: South Africa, E. Cape, Grahamstown).

##### Distribution

AF: South Africa (Grichanov 2009).

#### Euxiphocerus savannensis savannensis

Grichanov, 2009

2FC3F7E8-6635-57CD-BD22-DAECE42BF3EF

Euxiphocerus savannensis savannensis Grichanov, 2009: 130 (Type-locality: Namibia, Katima Mulilo Dist., Ndopu village).

##### Distribution

AF: Namibia (Grichanov 2009).

#### Euxiphocerus wulfi

Parent, 1935

29D7A0A6-0AC5-5EF3-925D-62EE8E7D0CC0

Euxiphocerus wulfi Parent, 1935: 122 (Type-locality: DR Congo, Rutshuru).

##### Distribution

AF: DR Congo (Grichanov 2009).

#### Euxiphocerus jinxiuensis

(Lin & Yang, 2022) comb. nov.

9B7707B5-0D7A-514D-9755-C1859D77BB98

Systenus jinxiuensis Lin & Yang, 2022: 294 (Type-locality: China, Guangxi, Jinxiu).

##### Distribution

OR: China (Lin and Yang 2022).

#### Euxiphocerus sinensis

(Yang & Gaimari, 2004) comb. nov.

44AEAD74-BB25-5B54-AADC-5D8DAC9F403A

Systenus sinensis Yang & Gaimari, 2004: 176 (Type-locality: China, Yunnan, Xishuangbanna).

##### Distribution

OR: China (Yang and Gaimari 2004).

## Identification Keys

### Key to species of male genus *Euxiphocerus* of worldwide

**Table:** 

1	Postocular setae flattened; male antennal pedicel greatly reduced; male postpedicel 5–6 times longer than high at base; male 7th abdominal segment short; hypopygium sessile, with large epandrial lobe, with broad and deeply divided dorsal and ventral arms of surstylus	2 (*Euxiphocerus* Parent, 1935)
–	Postocular setae simple; male antennal pedicel not reduced; male postpedicel at most 3–4 times longer than high at base; male 7th abdominal segment long, forming peduncle for hypopygium; epandrial lobe usually reduced to 2 pedunculate setae; dorsal and ventral arms of surstylus usually fused, with emargination at apex, or only ventral arm broad	*Systenus* Loew, 1857
2	Setae on head and thorax mainly pale white or pale brown	*E.disjuctus* Grichanov, 2009
–	Setae on thorax black or dark brown	3
3	Setae on head mainly pale white; arista swollen at apex	*E.sinensis* (Yang & Gaimari, 2004) comb. nov.
–	Setae on head mainly black or dark brown; arista normal	4
4	All coxae black	*E.jinxiuensis* (Lin & Yang, 2022) comb. nov.
–	At least fore coxa mainly yellow to pale yellow	5
5	Hind coxa mainly yellow to pale yellow	6
–	Hind coxa mainly black	7
6	All coxae with white setae; setae on abdomen mainly pale yellow to pale brown; dorsal lobe of surstylus without ventral projection	*E.lignicola* sp. nov.
–	All coxae with black setae; setae on abdomen mainly black; dorsal lobe of surstylus with ventral projection	*E.savannensiscapensis* Grichanov, 2009
7	Femora entirely yellow; epandrial lobe elongate-triangular, with pointed apex and a row of strong setae	*E.wulfi* Parent, 1935
–	Fore femur except distal 1/4 and mid femur in basal half black; epandrial lobe rounded-ovate, covered with setulae	*E.savannensissavannensis* Grichanov, 2009

Based on Grichanov (2009), Lin and Yang (2022) and Yang and Gaimari (2004)

## Supplementary Material

XML Treatment for Euxiphocerus

XML Treatment for Euxiphocerus lignicola

XML Treatment for Euxiphocerus jinxiuensis

XML Treatment for Euxiphocerus sinensis

XML Treatment for Euxiphocerus disjunctus

XML Treatment for Euxiphocerus lignicola

XML Treatment for Euxiphocerus savannensis capensis

XML Treatment for Euxiphocerus savannensis savannensis

XML Treatment for Euxiphocerus wulfi

XML Treatment for Euxiphocerus jinxiuensis

XML Treatment for Euxiphocerus sinensis
